# *KRAS* gene mutation quantification in the resection or venous margins of pancreatic ductal adenocarcinoma is not predictive of disease recurrence

**DOI:** 10.1038/s41598-022-07004-x

**Published:** 2022-02-22

**Authors:** Samuel Amintas, Benjamin Fernandez, Alexandre Chauvet, Laurence Chiche, Christophe Laurent, Geneviève Belleannée, Marion Marty, Etienne Buscail, Sandrine Dabernat

**Affiliations:** 1 Université de Bordeaux, 33000 Bordeaux, France; 2 Inserm U1312 «BoRdeaux Institute of onCology», BRIC, Team Biotherapy Genetics and Oncology, 33000 Bordeaux, France; 3 Centre Hospitalier Universitaire (CHU) de Bordeaux, 33000 Bordeaux, France; 4 Centre Hospitalier Universitaire (CHU) de Toulouse, 31000 Toulouse, France; 5 Inserm, UMR-1220, IRSD, 31000 Toulouse, France; 6 Université de Toulouse III, 31000 Toulouse, France

**Keywords:** Tumour biomarkers, Cancer, Pancreatic cancer

## Abstract

Pancreatic ductal adenocarcinoma (PDAC) patients eligible for curative surgery undergo unpredictable disease relapse. Even patients with a good pathological response after neoadjuvant treatment (NAT) remain susceptible to recurrent PDAC. Molecular analysis of R0 margins may identify patients with a worse prognosis. The molecular status of mutant *KRAS* (exon 2, codon 12/13) was analysed retrospectively by digital droplet PCR in tumour areas, venous and resection margins of resected tumours, either undergoing up-front surgery (UFS) or after NAT with a good pathological response. Expectedly, tumour tissues or remnants from patients who underwent NAT presented lower *KRAS* mutant allele frequencies (MAF) than patients eligible for UFS. Similarly, ypT1 tumour MAFs were greater than the ypT0 tumour remnant MAFs in the NAT group. Mutant *KRAS* status in margins did not distinguish NAT subgroups. It was not predictive of shorter recurrence-free or overall survival within or between groups. *KRAS*-double negativity in both venous and resection margins did not identify patients with a better prognosis, regardless of the groups. The cohorts ‘sizes were small due to limited numbers of patients meeting the inclusion criteria, but *KRAS*-positivity or MAFs in resection and venous margins did not carry prognostic value. Comparison of margins from good versus bad responders receiving NAT may provide better clinical value.

## Introduction

Pancreatic adenocarcinoma (PDAC) is lethal with limited efficacy of current treatments. Late diagnosis due to asymptomatic tumour development and unspecific symptoms contributes to the bad prognosis. A vast majority of patients (85%) suffer from locally advanced tumours and/or metastatic disease^1^.

Imaging at diagnosis is used to establish the tumour stage for non-metastatic patients. For this, contacts of the tumour with the mesenteric and hepatic vessels classify the tumour as resectable, borderline, and locally advanced^2^.

Surgery is the sole curative treatment but only 15% of patients present up-front resectable cancer. Moreover, even when resection is possible and complete, numerous patients relapse, without possible identification before surgery by predictive markers^3^. Two populations of patients can undergo surgical resection. First, patients eligible for up-front surgery carry tumours with no vascular contact. The second population is brought to the surgery by neoadjuvant therapy (NAT) of borderline and locally advanced diseases. NAT regimen includes chemotherapy (mainly FOLFIRINOX), with optional radiotherapy^4^, with better survival^5^ compared to up-front resectable patients^6^. Several recent trials have also demonstrated the benefit of adjuvant therapy. For example, PRODIGE 24 trial showed improvement in overall survival for patients treated with FOLFIRINOX compared to those treated with 5-FluoroUracil (5-FU, median overall survival of 54.4 months versus 35 months)^7^.

Current post-surgery tools are insufficient to both predict the course of the disease and estimate survival probability. For resected pancreatic cancers, tissue analysis carries prognostic value, in particular, the histological subtype and the presence of vascular and/or perineural invasion^8,9^. However, only 38% of T1/2N0R0 are disease-free 5 years after the diagnosis. In the same way, 28% of ypT0N0R0 tumours relapse 2 years after surgery^10^. Carcinogenic antigen 19–9 (CA19-9) has post-surgery prognostic value^11^–^13^. However, its prognostic value was originally established on heterogeneous populations receiving gemcitabine monotherapy while the standard of care has now evolved to combined regimens (FOLFIRINOX or gemcitabine + nab-paclitaxel). Up to 10% of the general population does not express this marker, leading to potential false negatives. CA19-9 may be falsely elevated with biliary obstruction, biliary endoprostheses, and/or cirrhosis. Drops in CA19-9 levels with current chemotherapies were predictive of longer progression-free survivals (PFS) underlining its utility as a follow-up marker more than a prognostic marker at the time of diagnosis^14^. Non-invasive follow-up of resected PDAC can be performed with a new companion tool, liquid biopsy. In particular, the quantification of circulating tumour DNA (ctDNA) or circulating tumour cells (CTCs), carries prognostic value^15^, but still needs improved sensitivity, especially for resectable patients who display less circulating tumour elements. By contrast, in the resected population, tumour material is available and may provide valuable molecular information that can relate to prognosis.

Mutations of the *KRAS* gene are present in 90–95% of pancreatic adenocarcinomas. It is the most frequent mutated gene in PDAC^16^. Constitutive activation of *KRAS* occurs after point mutations preferentially in the exons 12 and 13, which both represent > 98% of all activating mutations^17^. Furthermore, studies suggested that the molecular detection of *KRAS* mutations in surgery margins on histological samples could represent a sensitive way to identify residual disease^18^. *KRAS* mutation-positivity within the resection margins indicates that tumours cells may persist after resection. In the same way, molecular detection of *KRAS* mutations within the venous margins of the resected tumour may indicate tumour cell dissemination. Venous margins were analysed on a cohort of 22 patients with resectable PDAC naïve of neoadjuvant therapy, using a quantitative real-time polymerase chain reactive (Q-PCR) technique to detect *KRAS* mutations. Although all tumour displayed pathological R0 resection margins, 55% of the histologically venous margins displayed detectable *KRAS* mutations, associated with a worse prognosis as compared to KRAS-negative venous margins^19^. However, Q-PCR is less sensitive than droplet digital PCR (ddPCR), leaving the possibility of KRAS-positivity underestimation*.* More recently, KRAS mutational status within the resection margins of resected tumours from 81 patients with or without neoadjuvant therapy (NAT) was prognosis of recurrence-free survival^20^. However, the analysis included pathological R1 surgical resections, which may overestimate the recurrence rate. Thus, the detection of *KRAS* mutations within the venous and resection margins of resected PDAC tumours seems valuable. Nevertheless, both the venous and resection margins have not been analysed within the same tumours, using sensitive ddPCR.

This study aimed to investigate the presence of *KRAS* mutations by ultra-sensitive droplet digital PCR in the resection and venous margins of R0 resected tumour samples, identifying rare microscopically undetectable tumour cells in tissue areas, declared healthy by the pathologist. We hypothesized that *KRAS* mutation-positivity in resection or venous margins from patients receiving neoadjuvant therapy with good ypT0N0R0 pathological response and longer survival would be lower to undetectable, as compared to samples from resection and venous margins of up-front surgery R0 tumours, with shorter survival. In the same way, we also asked whether a lower molecular residual disease in the resection or venous margins identified patients with longer progression-free or overall survival, regardless of the resection history.

## Methods

### Patients’ inclusion

**Figure 1:**
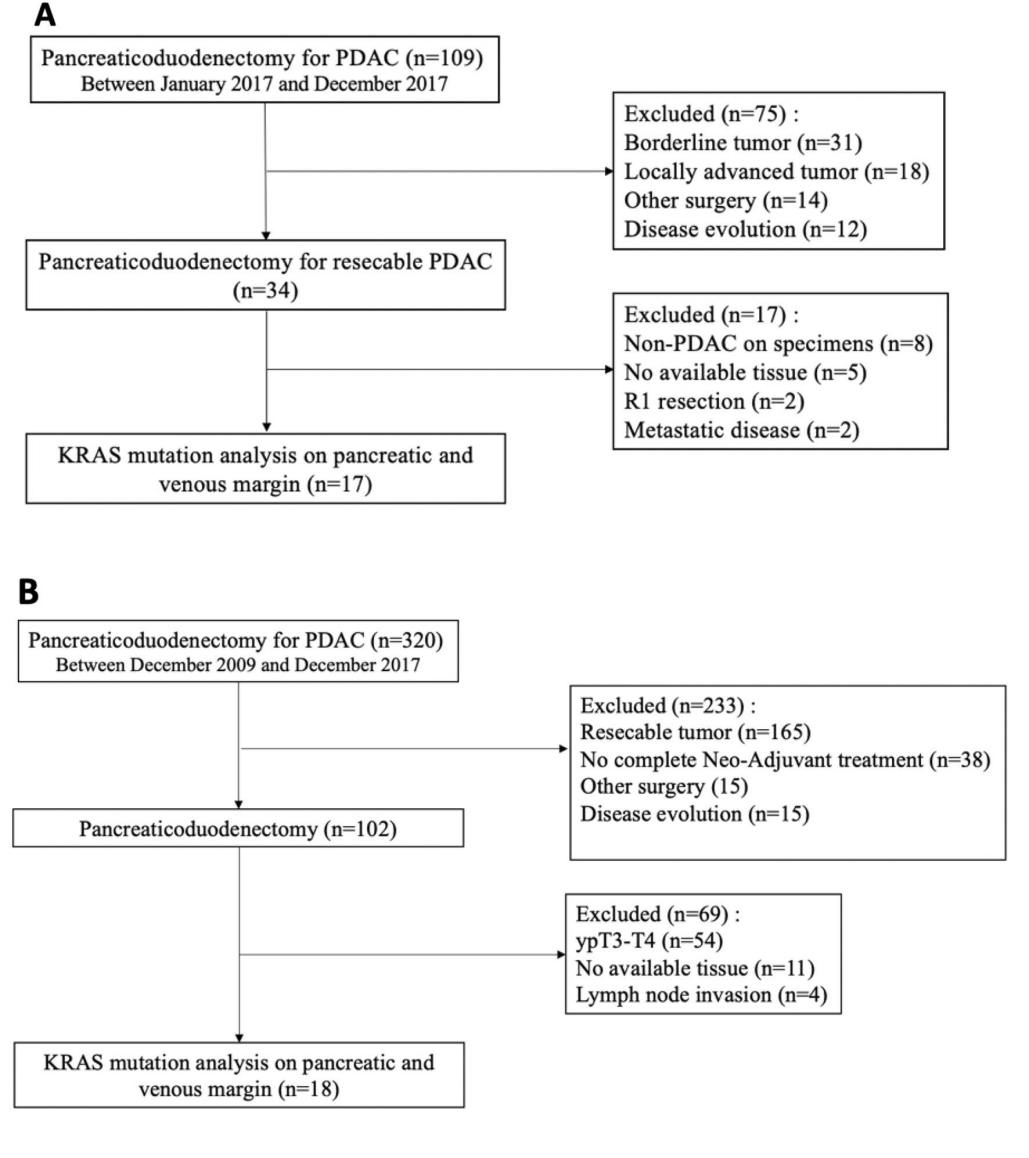
Flow chart of the two populations studied. (**A**) Up Font Surgery population (UFS, PANC-CTC #NCT03032913), (**B**) Neoadjuvant treatment (NAT) good responders. *PDAC: Pancreatic ductal adenocarcinoma.

For patients undergoing PDAC up-front surgery, cohort description was previously published^21,22^. Briefly, we enrolled patients with resectable pancreatic cancer (up-front surgery group, UFS, n = 22, Fig. 1A). Only the population of PDAC patients was studied here. The study was approved by the French Ministry of Research under the number 2016-A00431-50. The registered clinical trial (https://clinicaltrials.gov/ct2/show/NCT03032913?term=panc-ctc&draw=2&rank=1) was first published 2017–01-26 under the number NCT03032913. All patients provided written consent.

NAT good responders were selected retrospectively in the Bordeaux University Hospital Hepatobiliary Department database including all patients undergoing pancreaticoduodenectomy (PD) of pancreatic ductal adenocarcinomas. The database was started in 2009 and was prospectively incremented since. All patients provided written consents and the study was declared and approved by the Bordeaux University Hospital Ethics comity. Inclusion criteria were as follows: Patients with locally advanced and/or borderline pancreatic head tumour lesions undergoing surgery after neoadjuvant treatment, and displaying a ypT0/1N0 pathological response on the resected tumour residue (good responders, Fig. 1B).

## Pathological and molecular analyses

All methods were performed in accordance with the relevant guidelines and regulations, including French law and recommendations by providers.

Pathological margin status was determined by trained pathologists. All resection pieces underwent multicolored inking of the three resection margins (superior mesenteric vein / portal vein; superior mesenteric artherea; posterior margin). Resection margins were microscopically examined, and resection margin distance was measured from the nearest surgical resection margin to the tumour cells up to the submillimeter level. A margin was considered R0 when the tissue located at least 1 mm from the tumour limit was free of microscopically detectable tumour cells^23^.

Detection of mutant *KRAS* alleles was carried out on tumours (UFS) or tumour residues (NAT) and matched resection and venous margins. Histological selection of the 3 areas of interest, containing at least 50% of cells in the resection margins, and 20% of cells in the venous margins, was performed by trained pathologists. DNA of each area of interest was extracted from a 1 mm punch made in the paraffin block with the Maxwell RSC DNA FFPE Kit (Promega, Charbonnières-les-Bains, France). Extracted DNA was eluted in 70 µl of "nuclease-free" water and DNA concentration was determined by fluorimetry with DS11FX automated system (DeNovix), with final concentrations shown in Supplemental Tables 1–3. Digital droplet PCR was performed in duplicate on each DNA sample using the ddPCR *KRAS* Screening Multiplex Kit (Bio-Rad, Marnes-la-Coquette, France). The probes cover the 7 most common mutations of *KRAS* codons 12 and 13 (G12S, G12R, G12C, G12D, G12A, G12V, and G13D). Eight different probes are labeled either with a HEX fluorochrome (wild-type probe) or with a FAM fluorochrome (mutant probes). For each reaction, 50 ng of DNA, 10 µl of 2 × ddPCR Supermix for Probes (non dUTP), and 1 µl of 20X multiplex primers/probes (FAM + HEX) were used and mixed in a total volume of 20 µl. Droplets were generated with droplet generation oil for probes (Bio-Rad) within the QX200 Droplet Generator (Bio-Rad). Incubation of the droplets at 95 °C was performed for 10 min, followed by 40 cycles of PCR (denaturation at 94 °C for 30 s/annealing and elongation at 55 °C for 1 min) on a C-1000 Touch Thermal Cycler (Bio-rad). During all of the PCR steps, the increment of the temperature ramp is 2.5 °C. The droplet fluorescence analysis was carried out using a QX200 droplet reader (Bio-Rad) and data analysis with the Quantasoft Analysis Pro software (Bio-rad). All DNA samples had more than 10.000 valid droplets as recommended by the supplier. In the same way, the total numbers of DNA copies were over 5000 for all samples. Moreover, adequate DNA controls were used for each run: Blank (no template), WT (*KRAS*^*WT/WT*^ genomic DNA), Heterozygous (*KRAS*^*WT/G12D*^), and Homozygous (*KRAS*^*G12D/G12D*^) (Supplemental Fig. 1). To determine droplet status, thresholds for FAM and HEX fluorescence channels were set according to the control DNA samples. *KRAS* mutant droplets presented FAM fluorescence amplitude > 8000 (a.u.) and HEX fluorescence amplitude < 4000. *KRAS* WT droplets presented FAM fluorescence amplitude < 8000 and HEX fluorescence amplitude > 4000.

**Table 1:** Clinicopathologic characteristics of the study patients.

Characteristics	Neoadjuvant treatment group (n = 18)	Up-front surgery group (n = 17)
	No of patients (%)	No of patients (%)
**Age, y**		
≤ 60	4 (22)	2 (12)
> 60	14 (88)	15 (88)
**Sex**		
Male	12 (67)	10 (59)
Female	6 (33)	7 (41)
**Differentiation**		
Well	8 (44)	5 (29)
Moderate	7 (39)	7 (42)
Poor	3 (17)	5 (29)
**Lymph node invasion**		
Yes	0 (0)	17 (100)
No	18 (100)	0 (0)
**T category**		
T0	6 (33)	0
T1-T2	12 (67)	2 (12)
T3-4	0	15 (88)
**Adjuvant treatment**		
Yes	3 (17)	12 (71)
No	15 (83)	5 (29)
GEMCITABINE	2 (11)	4 (24)
mFOLFIRINOX*	1 (6)	13 (76)

*Modified Folfirinox.

Thirty-six *KRAS*^*WT/WT*^ controls samples were used for KRAS positivity threshold determination. They all carried WT KRAS exon 2, as sequenced by next-generation sequencing (NGS) using a custom panel using a custom Ion Ampliseq panel (Thermo Fisher Scientific, Life Technologies, Les Ullis, France). Libraries were amplified by emulsion PCR (polymerase chain reaction), enriched using automatic system Ion Chef automaton (Thermo Fisher Scientific), and then sequenced on 530 chips (ThermoFisher Scientific) using an Ion Torrent S5 sequencer (ThermoFisher Scientific) with a high average sequencing depth (greater than 1000X). Torrent Suite™ version 5.0 software (ThermoFisher Scientific) was used to perform data analysis. Reads were mapped to the human hg19 reference genome. Data processing, alignment, and mutation calling were performed using the Torrent Suite™. VCF files generated by Variant Caller were annotated by the ANNOVAR^24^. Molecular analysis (ddPCR and NGS) was carried out at Bordeaux University Hospital Somatic Molecular Biology Laboratory, according to the norm ISO15189, certifying clinical quality technical implementation.

## Follow-up

Patients underwent the outpatient visit 1 month after the surgery, then every 3 months for the first 2 years, and every 6 months thereafter. Recurrence-free survival (RFS) was defined as the time from resection of PDAC to the first radiologic recurrence or death due to PDAC. Overall survival (OS) was defined as the time from the diagnosis until death due to PDAC.

## Statistical analysis

Categorical variables were expressed as numbers and percentages. Continuous variables were expressed as means and standard deviations. A chi-squared test was used to compare categorical variables, and a non-parametric Mann–Whitney U test to compare continuous variables. Fisher’s exact test was used to compare the proportions of positive margins. Survival rates were calculated by the Kaplan–Meier method, and statistical significance was examined by the log-rank test and a Cox proportional hazards regression model. Outliers were detected by Grubbs’ test. All statistical analyses were performed using GraphPad Prism Version 5.03 (GraphPad Software, Inc., La Jolla, California, USA). *p* < 0.05 was considered statistically significant.

## Results

### Patient’s characteristics

The baseline characteristics of the cohorts (Fig. 1) and pathology data are shown in Table 1. Briefly, the mean age was 66 ± 8.2 years old in the NAT group and 62 ± 9.1 years old in the UFS group. In the NAT group, 33% of patients (n = 6) presented pathological complete response and 67% (n = 12) were classified T1 according to the eighth edition of the American Joint Committee on Cancer TNM staging system^25^. In the UFS group, most tumours were T3 or T4 (88% of cases, n = 15) and a minority were T2 (n = 2, 12%). Three patients (17%) received adjuvant chemotherapy in the NAT group versus 12 patients (71%) in the UFS group.

## Residual molecular disease in tumour tissues

We evaluated whether molecular quantification of mutant *KRAS* paralleled the pathological response in the NAT group or was predictive of worse prognosis in both groups.

**Figure 2:**
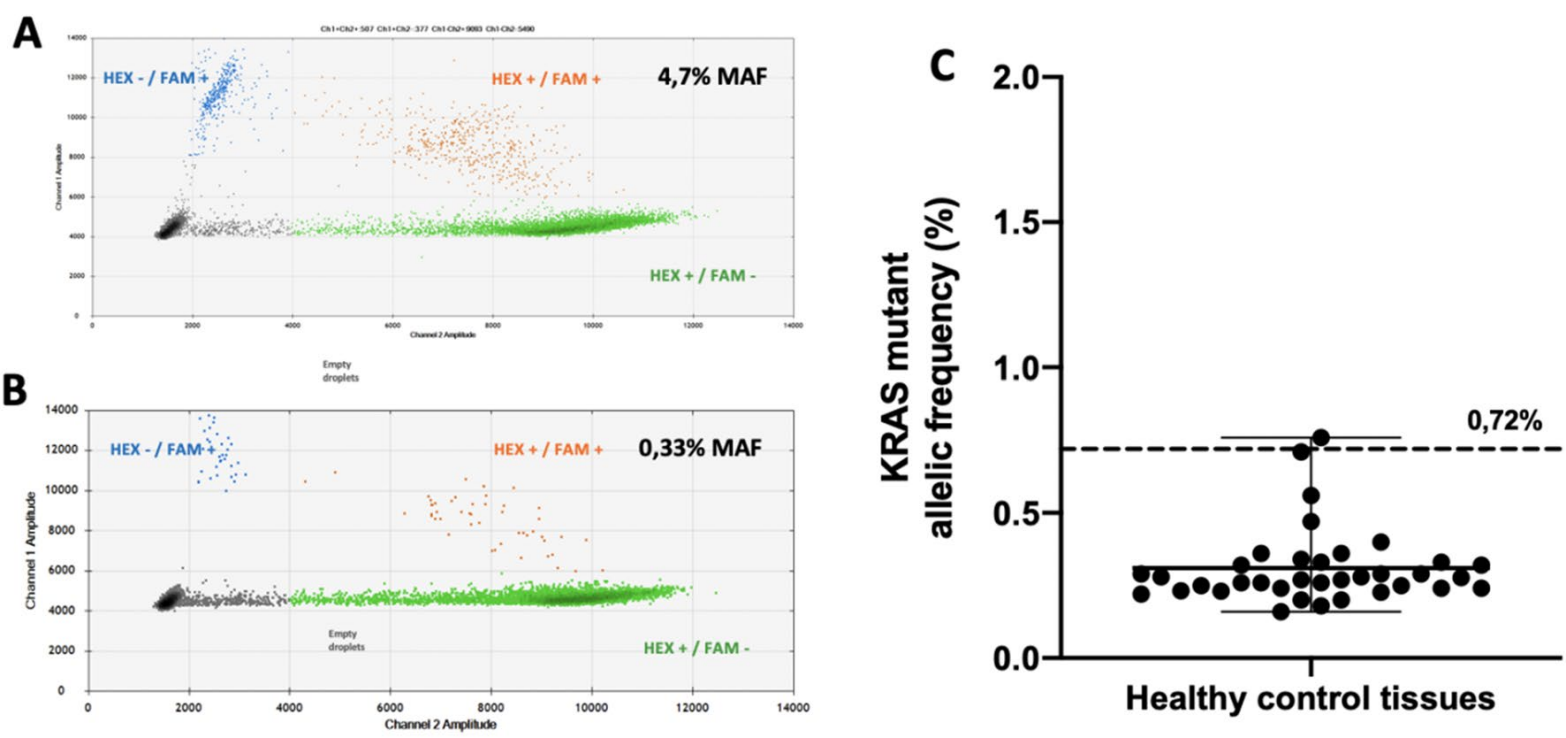
Droplet digital PCR control samples and assessment of molecular margin KRAS positivity threshold. Droplet digital PCR Two-dimensional (2D) plots (**A**) Margin DNA sample negative for KRAS mutation (FA = 0.33%) (**B**) Margin DNA sample positive for KRAS mutation (FA = 4.7%) (**C**) KRAS mutant allele frequency of the 36 KRAS wild type DNA controls (Mean = 0.31 ± 0.13% with range = 0.6%). The positivity threshold was calculated as the mean + 3 SD of MAFs of the 36 samples (0.72%).*MAF: Mutant allele frequency.

First, we determined that the *KRAS* mutant allele frequency (MAF) positivity threshold was 0.72%, using non-tumour DNA extracted from formol-fixed paraffin-embedded (FFPE) healthy tissue samples with proven *KRAS*^*WT*^ status by NGS. Indeed, formalin fixation and paraffin embedding can alter the quality of nucleic acids, such as cytosine deamination that can be amplified by PCR as thymines^26^. Moreover, as with any technique, ddPCR can generate false positives, which rate needs to be determined by running WT DNA treated/extracted exactly like the samples of interest, as recommended by the ddPCR kit provider. The positivity threshold was calculated as the mean + 3SD of MAFs (0.31 + 3X0.135%) from 36 healthy samples. *KRAS* mutational sample status was considered positive when the lower standard deviation value of each MAF was greater than the positivity threshold. Examples of positive and negative *KRAS* mutation results are presented in Figs. 2A and 2B.

**Figure 3:**
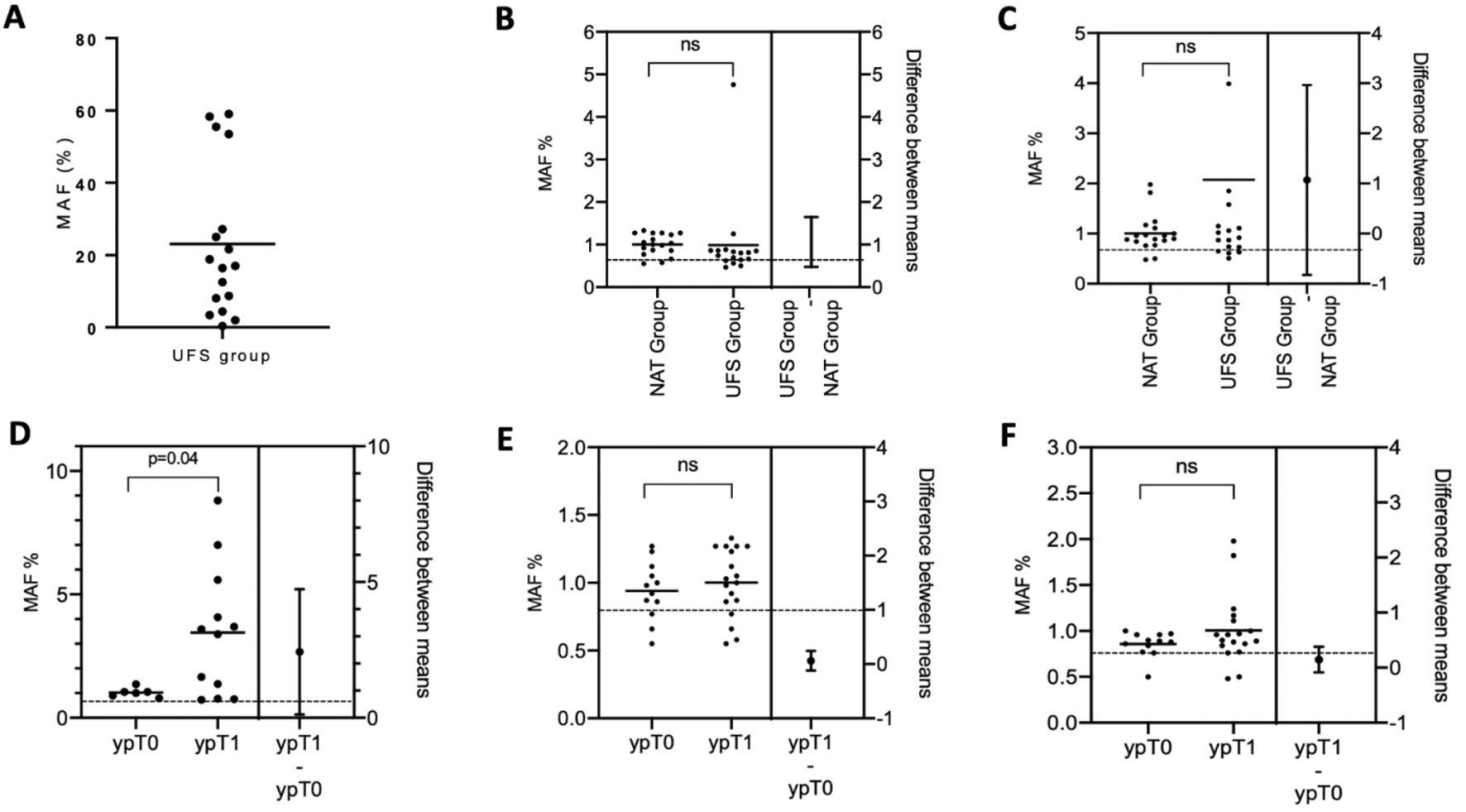
(**A**) Distribution of MAFs in tumour tissue from UFS group (line represents the average MAF value); Student t-test compared KRAS MAFs in sub-group of PDAC, (**B**) Resection margin between NAT and UFS group (*p* = 0.95), (**C**) Venous margin between NAT and UFS group (*p* = 0.26), (**D**) Tumour tissue between ypT0 and ypT1 tumour in the NAT Group (*p* = 0.04); (**E**) Resection margin between ypT0 and ypT1 (*p* = 0.30), (**F**) Venous margin between ypT0 and ypT1 (*p* = 0.14). *MAFs: Mutation allelic frequencies; NAT: Neoadjuvant treatment; UFS: Up-front surgery.

Next, we quantified *KRAS* MAFs in tumour tissues from patients who underwent UFS, and found, as expected a high average value of 23 ± 20.5% (Fig. 3A). In resected tumour tissues from patients receiving NAT, MAFs were significantly higher in the ypT1 group than the ypT0 group (average MAFs 3.5 ± 2.62% and 1.1 ± 0.2%, respectively, *p* = 0.04; Fig. 3B), as expected.

Thus, molecular quantification of mutant *KRAS* in tumour tissues is in agreement with expected results.

## Assessment of *KRAS-*positivity in resection and venous margins

Pathological R0 tumour resection or venous margins do not carry reliable prognostic value since the majority of patients undergo disease relapse^27^. Thus we hypothesized that considering the fibrotic nature of PDAC, the identification of residual tumour cells after NAT is very challenging for the pathologist. It is even more difficult after UFS since tumour cells might blend into the surrounding healthy tissue. The same difficulties might arise during venous margin pathological analysis. We tested the possibility to assess residual tumour cells in resection and venous margins by the molecular presence of *KRAS* mutations.

The average MAFs of UFS samples were 1.0 ± 0.99% and 1.15 ± 0.84% (*p* > 0.05) in the resected and venous margins, respectively, close to the molecular positivity threshold (Fig. 3B and 3C). Of note, we found 1 outlier in the venous margin, excluded from the analysis, showing a MAF similar to that of the matching tumour sample (17 and 18.8%, respectively), suggesting the puncture of tumour tissue. Positive margin percentages were 23% (4 out 17) and 50% (8 out 16) for the resection and venous margins, respectively. Only 2 patients were double positives on both margins (12.5%) and 6 double negatives (37.5%).

In the NAT group, the average MAF in the resected margins was 1.0 ± 0.25% and 11 out 18 samples (61%) were above the positivity threshold (Fig. 3E). *KRAS* positivity in venous margins was 1.01 ± 0.38% (*p* > 0.05 as compared to resected margins), and 11 out 18 (61%) were above the positivity threshold. The mean MAFs in the resection or venous margins were not different in the UFS and NAT groups (Fig. 3F). A majority of patients were double positives on both margins (10 patients, 55.5%) and 5 were double negatives (27.5%).

Thus, unlike what we expected, the patients undergoing NAT followed by resection presented more (resection margins) or similar (venous margins) mutant *KRAS* molecular marks than patients eligible for UFS. In the same way, MAF averages were similar in both groups of patients.

## Survival analysis based on residual molecular disease

We evaluated whether margin-positivity was associated with patients’ survival. The mean follow-up period was 33 ± 17 months (median = 27 months; range: 7–64 months). As expected, and because we selected NAT good responders, the NAT group showed longer recurrence-free survival (RFS) and overall survival (OS) as compared to the UFS group (58.9 vs 13.9 months, *p* < 0.01 and 64.4 vs 21.2 months, *p* < 0.01, respectively (Supplementary Fig. 2A and 2B).

Expectedly, in the UFS group, high *KRAS* MAFs in tumour tissues (superior to the average MAF value of 23.3%) tended to be associated with shorter recurrence-free survival (RFS, 6.7 months for MAFs > 23.3% versus 16.5 months for MAFs < 23.3%, *p* = 0.1, Supplementary Fig. 3A), even if the analysis did not reach significance probably because of the small size of the cohort. This trend was less marked for overall survival (OS, median survival of 21.2 months for MAFs > 23.3% versus 18.9 months for MAFs < 23.3%, *p* = 0.08, Supplementary Fig. 3B).

**Figure 4:**
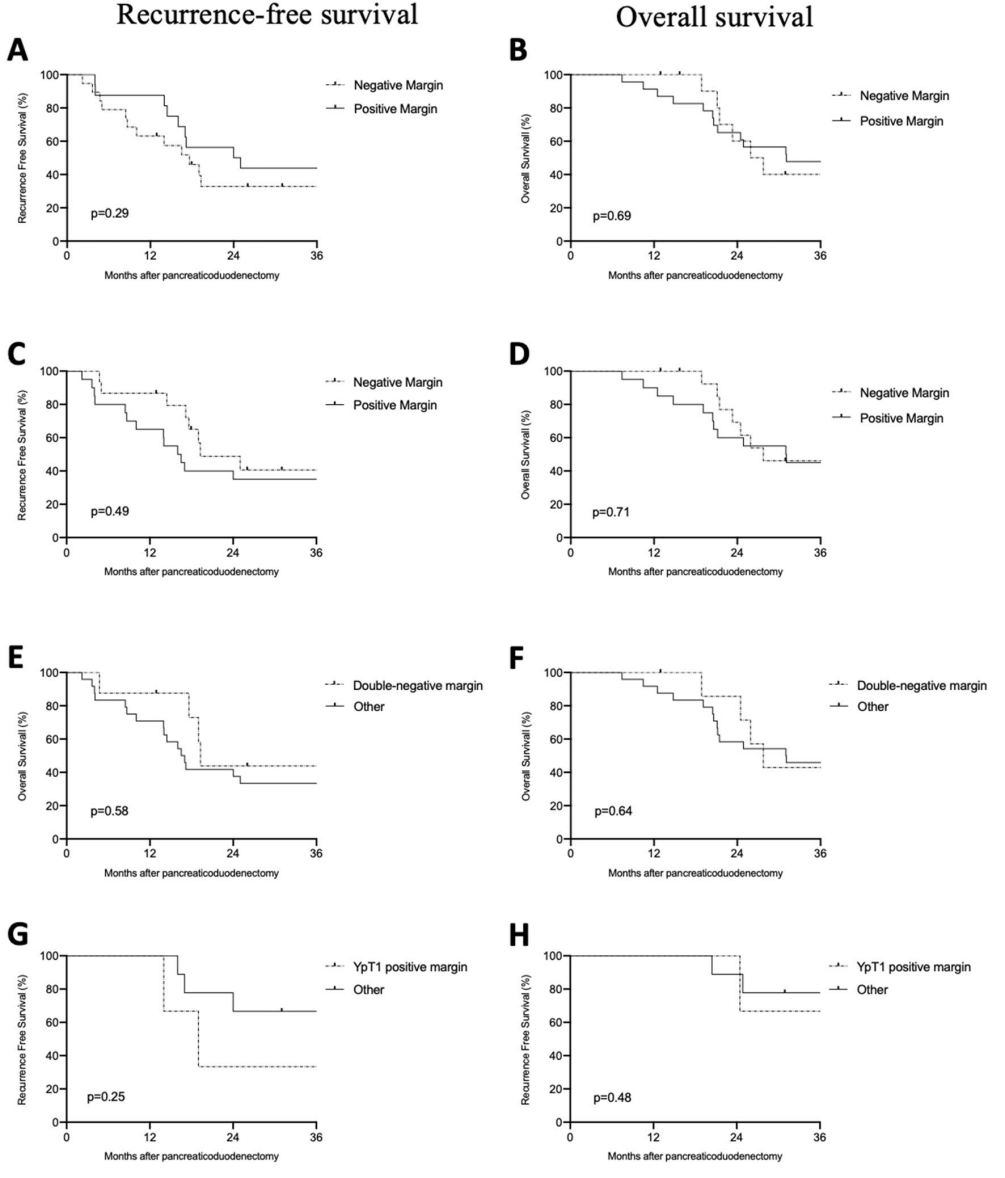
Kaplan–Meier survival analysis based on the molecular resection margin status. (**A**) Recurrence-free survival for resection margin, (**B**) Overall survival for resection margin, (**C**) Recurrence-free survival for venous margin, (**D**) Overall survival for venous margin, (**E**) Recurrence-free survival for double-negative margin, (**F**) Overall survival for double-negative margin, (**G**) Recurrence-free survival for ypT1venous and/or resection-positive margins, (**H**) Overall survival for ypT1venous and/or resection-positive margins.

Combined analysis of both groups showed no statistical difference in the RFS for positive versus negative resection margins (*p* = 0.29; Fig. 4A) or for positive versus negative venous margins (*p* = 0.49; Fig. 4C). In the same way, negative margins did not correlate with longer OS (Fig. 4B and 4D, p = 0.69 for resection margins and *p* = 0.71 for venous margins).

Double negative margins (i.e. patients for whom both margins were negative, excluding 3 patients with *KRAS*-negative tumours) did not correlate with longer RFS (Fig. 4E, p = 0.58) or OS (Fig. 4F, p = 0.64).

Finally, in the NAT group, double pathological (ypT1) and margin (either resection or venous) positivity did not identify patients with shorter RFS (Fig. 4G, n = 12, *p* = 0.25) or OS (Fig. 4H, p = 0.48), as compared to single positives.

Together, these results show that the detection of molecular residual disease in the resection or venous margins of UFS or NAT PDAC patients does not carry prognostic value.

In the same way, double-positive margins (resection and venous) or double-negative margins did not correlate with local or distant recurrence.

## Discussion

The present study tested the hypothesis that despite the R0 status of resected PDACs, residual disease predictive of disease relapse might be evidenced in the resected tissues by molecular identification of mutant *KRAS*. We examined the mutant *KRAS* status in tissue samples of patients with the best prognosis, i.e. the margins of R0 resected tissues of patients who underwent UFS and of NAT good responder patients who had surgery.

Even if *KRAS* is mutated in > 90% of PDACs, the use of *KRAS* mutation status as a prognostic marker largely depends on the capacity of the technique to detect *KRAS* mutant variants. Indeed, mutations occur preferentially in the G12 position (98%) while G13 and Q61 positions count for 1% each^17^ of all *KRAS* mutations. The G12 amino acid substitutions arise from 6 variants. In our study, 86% (25/29) of the DNAs from resected tissues, in which tumour remnants were expected (i.e. tumours of the UFS group and tumours of the ypT1 NAT sub-group), showed detectable *KRAS* mutations. Of note, 83% (5/6) of the ypT0 NAT sub-group displayed *KRAS* mutations, although the primary tumours were supposedly cleared. Overall, we found 1 UFS patient, 1 ypT0 patient, and 1 ypT1 patient who were triple-negatives (86%). This observation is in agreement with *KRAS* mutation frequency in PDAC (90–95% according to the studies, (reviewed in^28^) combined with the capacity of our technique to detect 98% of the mutations (all G12 and G13 codons substitutions). Altogether, these observations suggest that even if tumour clearance is pathologically confirmed, *KRAS* mutations persist despite clearing treatments. Although poorly described, micrometastases are supposed to exist, as early as at the onset of the disease. Indeed, PDAC can produce circulating tumour cells (CTCs) with metastatic potential during the formation of the primary tumour, before malignancy can be detected by histological methods^29^.

Patients with *KRAS* alterations have decreased OS, and specifically, the *KRAS* G12D mutation confers a worse prognosis in comparison with other *KRAS* alterations^28,30^. It would be interesting to determine *KRAS* mutation types in our cohorts and evaluate the link with prognosis.

The main limit of our study is the small size of the cohorts. However, they are representative of bigger published cohorts, in terms of RFS and OS. Indeed, in agreement with published resected cohorts comparable to our UFS group (for example^31^, with an OS of 22.1 months and RFS 11.0 months versus 21.2 months and 13.9 months, our study). The NAT group selected good responders (complete pathological response, ypT0/1/N0). Of all the patients examined during the period, they represented 5.6%, which is in the same range as previously observed^32^. NAT yielded good performance (8 out 18, i.e. 44% of relapses, and an RFS and OS of 58.9 and 64.4 months, respectively), as expected in published results^33^. In addition, our cohort yielded 17.6% of ypT0/1/N0 after chemoradiotherapy, which is in agreement with published data^33^. Finally, as expected, RFS and OS in the UFS group were lower than in the NAT group. However, the NAT group selected patients with good response (ypT0/1/N0) to treatment, which enhanced artificially the difference in the prognosis compared to the UFS group recruited prospectively. We also limited our analysis to the good responders of NAT, and it would be of interest to assess the margins of the ypT3/4/N0/N^+^.

Residual disease detection using ultra-sensitive ddPCR has routine applications for solid and haematological tumours assessment in plasma samples, with prognosis information^34^–^36^. *KRAS* mutational status was determined in the UFS cohort circulating plasma DNA (ctDNA)^21^. None of the resected patients had detectable circulating MAFs. Only 2 metastatic patients, excluded in this study, were found positive. As the NAT cohort was analysed retrospectively, we did not possess plasma samples and could not analyze the ctDNA. This is a limit of this study but, according to the longer survival of the NAT group, we can suppose that liquid biopsies would be negative. This important point deserves further validation.

Moreover, ddPCR is routinely used to detect targeted mutations in tumour tissues^37^. Residual disease evaluated by *KRAS* mutation positivity in margins was previously evaluated. In the venous margins of 22 UFS patients, *Turrini *et al*.* detected *KRAS* mutations in 55% of histologically negative venous margins^19^, which is similar to our finding here of 47% (8 out of 17) and *Kim *et al*.*’s^18^. However, by contrast to the cited studies, our survival analysis did not stratify patients according to their margin positivity. Adjuvant treatment cannot explain survival discrepancies. Interestingly, Kim’s retrospective cohort counted only 11% of T3 tumours for 89% of T1/T2 and 57% of N^+^. In the same way, Turrini’s patients were mainly T1/T2 (72.8%), and 59% N^+^ were recruited retrospectively. Our UFS prospective cohort included 88% of T3 tumours and only 12% of T1/T2 tumours, for 100% N^+^. Thus, it seems that Kim and Turrini’s cohorts are not comparable to ours, at least in terms of tumour grade, which might explain the different prognosis value of *KRAS* mutation presence in margins, which may be of clinical value for lower grade PDACs. More recently, *Kim *et al. found that patients showing a *KRAS* MAFs > 4.19% had shorter DFS and OS in resection margins, regardless of R status. They included R1 margins (43%), and it is not clear whether the 4.19% threshold was distinct from the positivity threshold^20^, since they did not detail this point. This high threshold may identify the R1 margins, hence its prognostic value. We rarely detected MAFs > 4.19% (2 out of 70 determinations), probably because we did not include R1 margins. Kim’s patients had 73% of T1/T2 tumours and 73% of N^+^. It would be interesting to analyze separately the 27% of patients with higher tumour grades.

## Conclusion

This work clarifies the prognosis significance of *KRAS* mutation-positivity in margins of resected tumours, using ultra-sensitive detection of KRAS mutations. When margins are positive for T1/T2/N0 tumours, it may carry prognostic value. T3/N^+^ tumours may be already too invasive for *KRAS*-positivity to distinguish worse prognosis. Overall, very few studies evaluated *KRAS* mutant margin positivity and small cohorts were tested. More prospective analyses, including the future NAT standard, need to be carried out. Our results are important to implement the knowledge of residual disease detection in PDAC resected samples. Finally, unlike hematological cancers, for which molecular residual disease can be followed-up by combined ddPCR and liquid biopsy, solid tumours, in particular, PDACs still need the development of sensitive molecular tools and/or the identification circulating biomarkers.

## Supplementary Information

Supplementary Information 1.

Supplementary Information 2.

Supplementary Information 3.

Supplementary Information 4.

Supplementary Information 5.
