# Supplementary material for: KRAS gene mutation quantification in the resection or venous margins of pancreatic ductal adenocarcinoma is not predictive of disease recurrence
Source: Sci Rep. 2022 Feb 22;12:2976. doi: 10.1038/s41598-022-07004-x (PMC8864048; doi:10.1038/s41598-022-07004-x)
Supplement: Supplementary file 1 — Supplementary Information 1. [file 41598_2022_7004_MOESM1_ESM.doc]

**Supplemental figure 1.** Droplet digital PCR control samples. Droplet digital PCR Two-dimensional (2D) plots **A**: Blank sample (no DNA input) **B**: KRAS G12/13 Wild-Type DNA sample **C**: KRAS G12/13 heterozygous DNA sample **D**: KRAS G12/13 homozygous DNA sample. *MAF: Mutant allele frequency

**Supplemental Figure 2.** Kaplan-Meier survival analysis comparing NAT and UFS groups. **A**: Recurrence-Free Survival, **B**: Overall Survival. * NAT: Neoadjuvant treatment; UFS: Up-front surgery

**Supplemental Figure 3.** Kaplan-Meier survival analysis comparing patients with tumour MAFs ≤ 23.3% and MAFs >23.3%. **A**: Recurrence-Free Survival, **B**: Overall Survival. * NAT: Neoadjuvant treatment; UFS: Up-front surgery
