# Supplementary figures and images for: KRAS gene mutation quantification in the resection or venous margins of pancreatic ductal adenocarcinoma is not predictive of disease recurrence
Source: Sci Rep. 2022 Feb 22;12:2976. doi: 10.1038/s41598-022-07004-x (PMC8864048; doi:10.1038/s41598-022-07004-x)

S Fig 1

**A**

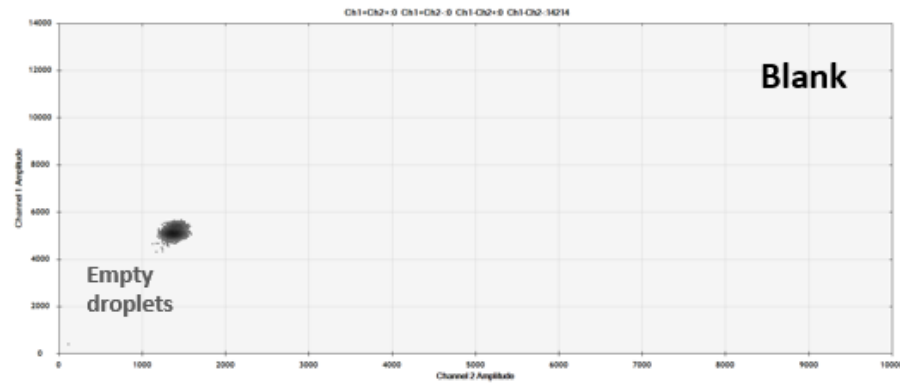

**B**

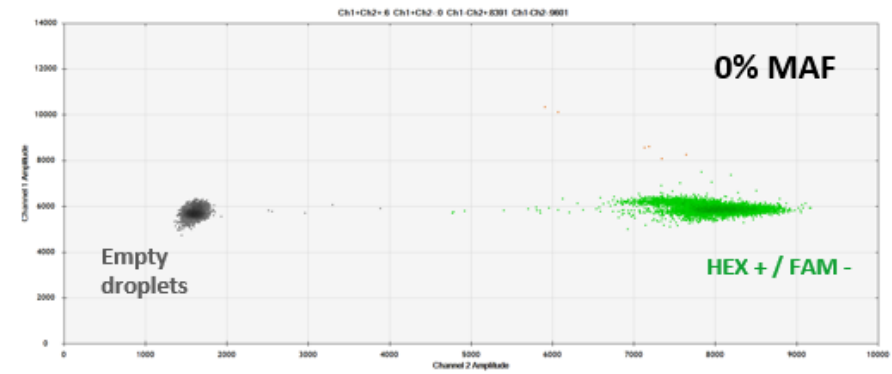

**C**

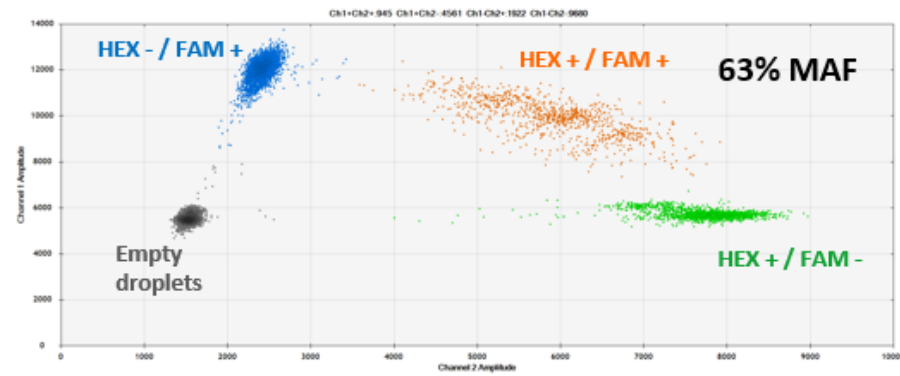

**D**

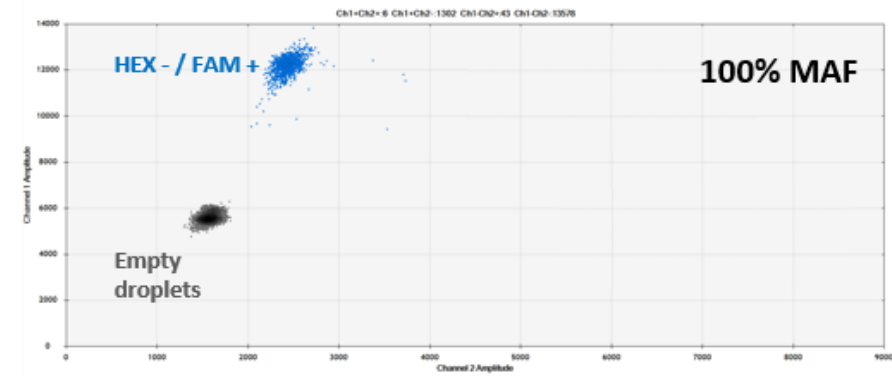

S Fig 2

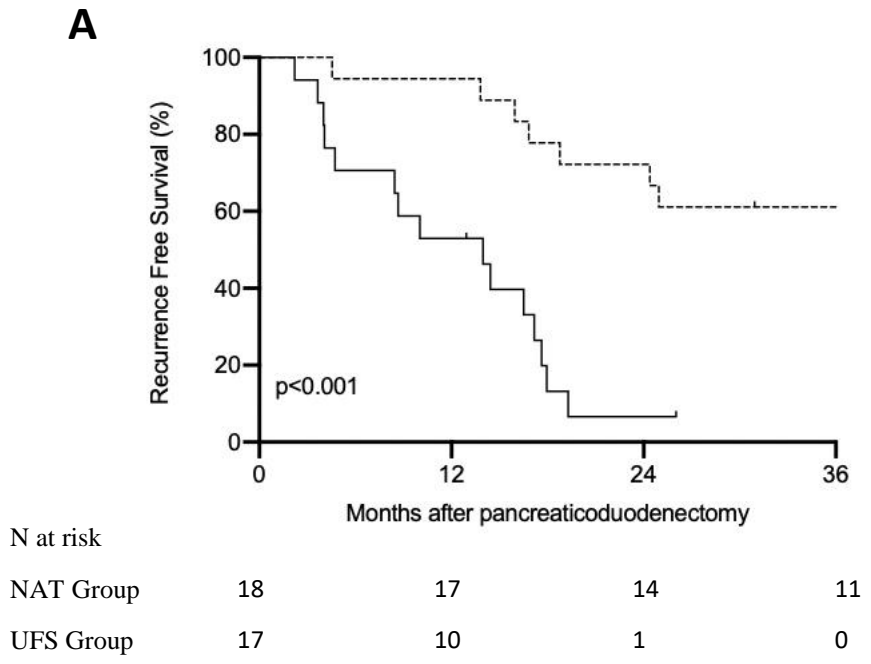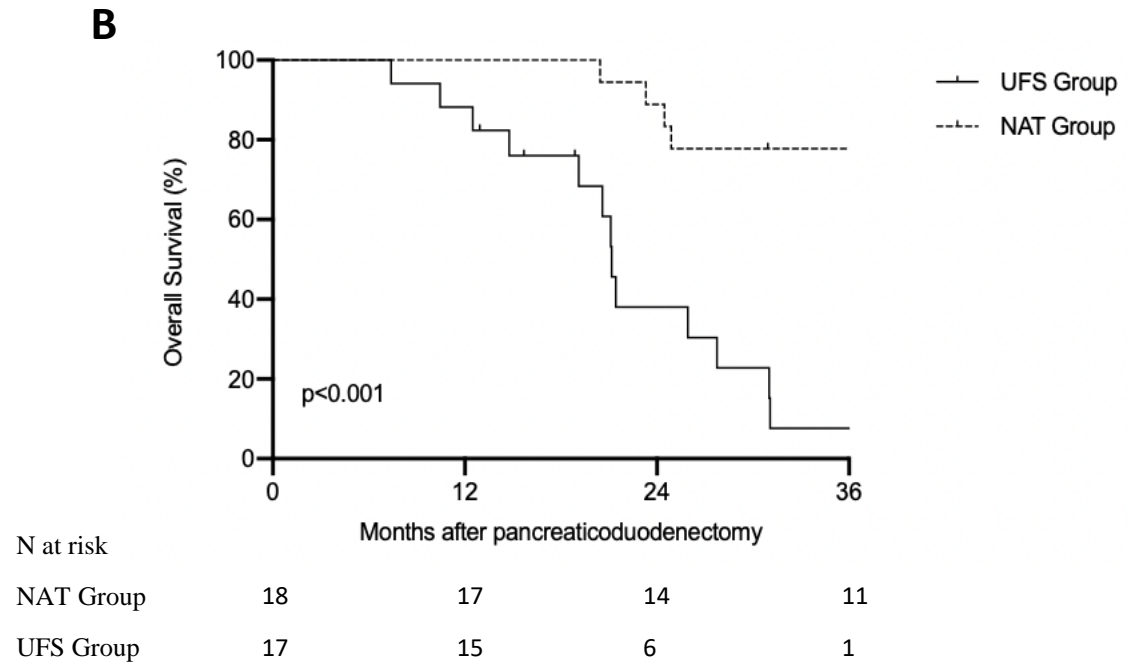

S Fig 3

**A**

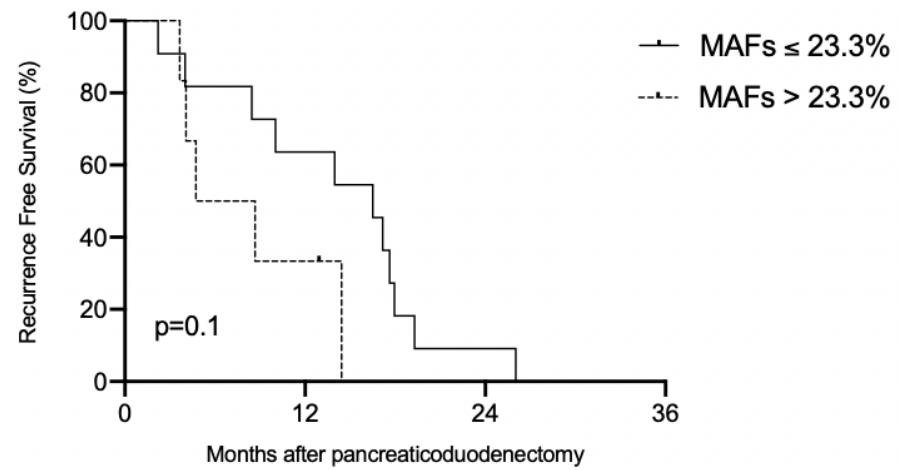

**B**

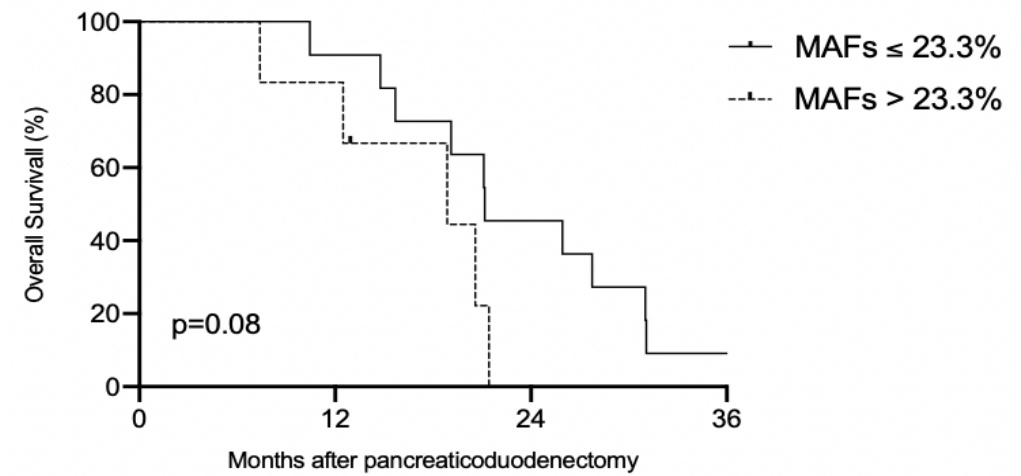

Supplement: Supplementary file 2 — Supplementary Information 2. [file 41598_2022_7004_MOESM2_ESM.pdf]
