# Supplementary material for: KRAS gene mutation quantification in the resection or venous margins of pancreatic ductal adenocarcinoma is not predictive of disease recurrence
Source: Sci Rep. 2022 Feb 22;12:2976. doi: 10.1038/s41598-022-07004-x (PMC8864048; doi:10.1038/s41598-022-07004-x)
Supplement: Supplementary file 3 — Supplementary Information 3. [file 41598_2022_7004_MOESM3_ESM.docx]

**Supplemental Table 1: DNA concentrations and MAFs for healthy samples**

| **Control tissue** | **DNA concentration (ng/µl)** | **KRAS MAF (%) with standard deviation** |
| --- | --- | --- |
| Healthy control tissue 1 | **170** | 0,2 (0,11 - 0,29) |
| Healthy control tissue 2 | **41** | 0,36 (0,24 - 0,48) |
| Healthy control tissue 3 | **77** | 0,36 (0,25 - 0,47) |
| Healthy control tissue 4 | **16** | 0,71 (0,59 - 0,83) |
| Healthy control tissue 5 | **24** | 0,28 (0,21 - 0,35) |
| Healthy control tissue 6 | **130** | 0,28 (0,21 - 0,35) |
| Healthy control tissue 7 | **62** | 0,33 (0,26 - 0,4) |
| Healthy control tissue 8 | **24** | 0,76 (0,63 - 0,89) |
| Healthy control tissue 9 | **29** | 0,56 (0,43 - 0,69) |
| Healthy control tissue 10 | **78** | 0,32 (0,2 - 0,44) |
| Healthy control tissue 11 | **54** | 0,32 (0,21 - 0,43) |
| Healthy control tissue 12 | **35** | 0,25 (0,18 - 0,32) |
| Healthy control tissue 13 | **17** | 0,27 (0,18 - 0,36) |
| Healthy control tissue 14 | **89** | 0,24 (0,16 - 0,32) |
| Healthy control tissue 15 | **156** | 0,34 (0,27 - 0,41) |
| Healthy control tissue 16 | **123** | 0,28 (0,22 - 0,34) |
| Healthy control tissue 17 | **132** | 0,29 (0,19 - 0,39) |
| Healthy control tissue 18 | **77** | 0,2 (0,12 - 0,28) |
| Healthy control tissue 19 | **45** | 0,22 (0,17 - 0,27) |
| Healthy control tissue 20 | **90** | 0,16 (0,12 - 0,2) |
| Healthy control tissue 21 | **62** | 0,24 (0,11 - 0,37) |
| Healthy control tissue 22 | **76** | 0,25 (0,19 - 0,31) |
| Healthy control tissue 23 | **45** | 0,27 (0,18 - 0,36) |
| Healthy control tissue 24 | **32** | 0,18 (0,13 - 0,23) |
| Healthy control tissue 25 | **87** | 0,23 (0,17 - 0,29) |
| Healthy control tissue 26 | **113** | 0,26 (0,14 - 0,38) |
| Healthy control tissue 27 | **89** | 0,23 (0,13 - 0,33) |
| Healthy control tissue 28 | **47** | 0,24 (0,17 - 0,31) |
| Healthy control tissue 29 | **32** | 0,26 (0,21 - 0,31) |
| Healthy control tissue 30 | **19** | 0,33 (0,27 - 0,39) |
| Healthy control tissue 31 | **78** | 0,23 (0,19 - 0,27) |
| Healthy control tissue 32 | **65** | 0,26 (0,15 - 0,37) |
| Healthy control tissue 33 | **24** | 0,29 (0,21 - 0,37) |
| Healthy control tissue 34 | **90** | 0,29 (0,24 - 0,34) |
| Healthy control tissue 35 | **57** | 0,4 (0,25 - 0,55) |
| Healthy control tissue 36 | **143** | 0,47 (0,39 - 0,55) |
