# Supplementary material for: KRAS gene mutation quantification in the resection or venous margins of pancreatic ductal adenocarcinoma is not predictive of disease recurrence
Source: Sci Rep. 2022 Feb 22;12:2976. doi: 10.1038/s41598-022-07004-x (PMC8864048; doi:10.1038/s41598-022-07004-x)
Supplement: Supplementary file 4 — Supplementary Information 4. [file 41598_2022_7004_MOESM4_ESM.docx]

**Supplemental Table 2: DNA concentrations and MAFs for NAT samples**

| **Patient number** | **Area of interest** | **DNA concentration (ng/µl)** | **KRAS MAF (%) with standard deviation** |
| --- | --- | --- | --- |
| Patient GR 1 | Tumor | **60** | 5.43 (5,07 – 5,79) |
|  | Resection margin | **28** | 0,91 (0,75 -1,07) |
|  | Venous margin | **68** | 0,96 (0,76 -1,16) |
| Patient GR 2 | Tumor | **74** | 1,06 (0,89 – 1,23) |
|  | Resection margin | **52** | 1,12 (0,9 -1,34) |
|  | Venous margin | **137** | 0,86 (0,66 -1,06) |
| Patient GR 3 | Tumor | **43** | 1.15 (0,94 – 1,36) |
|  | Resection margin | **24** | 1,23 (1 -1,46) |
|  | Venous margin | **17** | 0,89 (0,66 -1,12) |
| Patient GR 4 | Tumor | **17** | 1.05 (0,88- 1,22) |
|  | Resection margin | **20** | 1 (0,88 -1,12) |
|  | Venous margin | **22** | 0,98 (0,84 -1,12) |
| Patient GR 5 | Tumor | **50** | 0.9 (0,75 – 1,05) |
|  | Resection margin | **17** | 0,87 (0,69 -1,05) |
|  | Venous margin | **54** | 0,88 (0,7 -1,06) |
| Patient GR 6 | Tumor | **40** | 0,98 (0,78 – 1,1!) |
|  | Resection margin | **10** | 0,98 (0,65 -1,31) |
|  | Venous margin | **43** | 1,01 (0,11 -1,91) |
| Patient GR 7 | Tumor | **55** | 1.23 (1,06 – 1,4) |
|  | Resection margin | **34** | 0,66 (0,51 -0,81) |
|  | Venous margin | **16** | 0,7 (-0,4 -1,8) |
| Patient GR 8 | Tumor | **51** | 0.74 (0,61 – 0,87) |
|  | Resection margin | **37** | 0,55 (0,43 -0,67) |
|  | Venous margin | **48** | 0,76 (0,6 -0,92) |
| Patient GR 9 | Tumor | **97** | 3,49 (3,14 – 3,74) |
|  | Resection margin | **79** | 0,58 (0,45 -0,71) |
|  | Venous margin | **61** | 0,61 (0,5 -0,72) |
| Patient GR 10 | Tumor | **77** | 1.6 (1,4 – 1,8) |
|  | Resection margin | **22** | 1,2 (1,01 -1,39) |
|  | Venous margin | **46** | 0,89 (0,72 -1,06) |
| Patient GR 11 | Tumor | **13** | 8.34 (7,84 – 8, 84) |
|  | Resection margin | **3** | 1,14 (0,88 -1,4) |
|  | Venous margin | **19** | 1,44 (1,03 -1,85) |
| Patient GR 12 | Tumor | **14** | 0.74 (0,58 – 0,9) |
|  | Resection margin | **6** | 0,95 (0,76 -1,14) |
|  | Venous margin | **12** | 0,98 (0,82 -1,14) |
| Patient GR 13 | Tumor | **23** | 0.73 (0,58 – 0,88) |
|  | Resection margin | **39** | 0,84 (0,69 -0,99) |
|  | Venous margin | **68** | 0,77 (0,63 -0,91) |
| Patient GR 14 | Tumor | **50** | 0.8 (0,67 – 0,93) |
|  | Resection margin | **38** | 0,91 (0,76 -1,06) |
|  | Venous margin | **71** | 0,94 (0,77 -1,11) |
| Patient GR 15 | Tumor | **34** | 3.47 (3,19 – 3,75) |
|  | Resection margin | **66** | 1,21 (1,04 -1,38) |
|  | Venous margin | **15** | 0,97 (0,8 -1,14) |
| Patient GR 16 | Tumor | **94** | 3.56 (3,24 – 3,88) |
|  | Resection margin | **88** | 1,27 (1,04 -1,5) |
|  | Venous margin | **26** | 1,11 (0,91 -1,31) |
| Patient GR 17 | Tumor | **11** | 3.39 (2,89 – 3,89) |
|  | Resection margin | **186** | 1,27 (1 -1,54) |
|  | Venous margin | **16** | 1,98 (1,47 -2,49) |
| Patient GR 18 | Tumor | **26** | 7,06 (6,56 – 7,56) |
|  | Resection margin | **20** | 1,03 (0,85 -1,21) |
|  | Venous margin | **33** | 1,08 (0,8 -1,36) |
