# Supplementary material for: KRAS gene mutation quantification in the resection or venous margins of pancreatic ductal adenocarcinoma is not predictive of disease recurrence
Source: Sci Rep. 2022 Feb 22;12:2976. doi: 10.1038/s41598-022-07004-x (PMC8864048; doi:10.1038/s41598-022-07004-x)
Supplement: Supplementary file 5 — Supplementary Information 5. [file 41598_2022_7004_MOESM5_ESM.docx]

**Supplemental Table 3: DNA concentrations and MAFs for UFS samples**

| **Patient number** | **Area of interest** | **DNA concentration (ng/µl)** | **KRAS MAF (%) with standard deviation** |
| --- | --- | --- | --- |
| Patient UFS 1 | Tumor | **39,4** | 18,80 (18,5 – 19,1) |
|  | Resection margin | **4,6** | 0,64 (0,53 – 0,75) |
|  | Venous margin | **14** | 17,00 (16,5 – 17,5) |
| Patient UFS 2 | Tumor | **77** | 1.97 (1,72 – 2,23) |
|  | Resection margin | **7** | 0.83 (0,7 – 0,96) |
|  | Venous margin | **14** | 1,09 (0,89 – 1,29) |
| Patient UFS 3 | Tumor | **65** | 4,42 (4,18 – 4,66) |
|  | Resection margin | **28** | 0,64 (0,54 – 0,74) |
|  | Venous margin | **39** | 0,73 (0,61 - 0,85) |
| Patient UFS 4 | Tumor | **215** | 8.7 (8,26 – 9,14) |
|  | Resection margin | **14** | 0.8 (0,69 – 0,91) |
|  | Venous margin | **19** | 1.06 (0,74 – 1,38) |
| Patient UFS 5 | Tumor | **102** | 59,00 (58,2 – 59,8) |
|  | Resection margin | **13** | 0.74 (0,57 – 0,91) |
|  | Venous margin | **13** | 0.74 (0,62 – 0,74) |
| Patient UFS 6 | Tumor | **54** | 8.04 (7,82 – 8,3) |
|  | Resection margin | **40** | 0.88 (0,75 – 1,01) |
|  | Venous margin | **27** | 0.92 (0,81 – 1,03) |
| Patient UFS 7 | Tumor | **40** | 16.4 (16,1 – 16,7) |
|  | Resection margin | **25** | 0.81 (0,68 – 0,94) |
|  | Venous margin | **15** | 1.15 (0,91 – 1,39) |
| Patient UFS 8 | Tumor | **49** | 25,00 (24,3 – 25,7) |
|  | Resection margin | **24** | 0.85 (0,72 – 0,85) |
|  | Venous margin | **15** | 0.87 (0,69 – 1,05) |
| Patient UFS 9 | Tumor | **73** | 55.5 (54,2 – 56,8) |
|  | Resection margin | **20** | 0.56 (0,47 – 0,65) |
|  | Venous margin | **42** | 1.85 (1,65 – 2,01) |
| Patient UFS 10 | Tumor | **178** | 58.3 (57 – 59,5) |
|  | Resection margin | **87** | 4.76 (4,45 – 5,07) |
|  | Venous margin | **18** | 0.87 (0,71 – 1,03) |
| Patient UFS 11 | Tumor | **170** | 3.4 (3,16 – 3,64) |
|  | Resection margin | **57** | 0.66 (0,56 – 0,76) |
|  | Venous margin | **16** | 3.99 (3,57 – 4,41) |
| Patient UFS 12 | Tumor | **72** | 0.35 (0,29 – 0,41) |
|  | Resection margin | **13** | 1.25 (1,09 – 1,41) |
|  | Venous margin | **32** | 1.02 (0,88 – 1,16) |
| Patient UFS 13 | Tumor | **80** | 12.5 (11,8 – 13,2) |
|  | Resection margin | **23** | 0.5 (0,42 – 0,58) |
|  | Venous margin | **33** | 0.61 (0,53 - 0,67) |
| Patient UFS 14 | Tumor | **112** | 17,00 (16,5 – 17,5) |
|  | Resection margin | **25** | 0.86 (0,71 – 1,01) |
|  | Venous margin | **18** | 1.58 (1,42 – 1,74) |
| Patient UFS 15 | Tumor | **126** | 27.2 (26,8 – 27,6) |
|  | Resection margin | **38** | 0.69 (0,57 – 0,81) |
|  | Venous margin | **19** | 0.51 (0,31 – 0,71) |
| Patient UFS 16 | Tumor | **145** | 21.6 (21 – 22,2) |
|  | Resection margin | **35** | 0.47 (0,37 – 0,57) |
|  | Venous margin | **36** | 0.6 (0,53 – 0,67) |
| Patient UFS 17 | Tumor | **180** | 53.5 (52,9 – 54,1) |
|  | Resection margin | **10** | 0.86 (0,71 – 1,01) |
|  | Venous margin | **19** | 0.65 (0,55 – 0,75) |
